# FFAE-UNet: An Efficient Pear Leaf Disease Segmentation Network Based on U-Shaped Architecture

**DOI:** 10.3390/s25061751

**Published:** 2025-03-12

**Authors:** Wenyu Wang, Jie Ding, Xin Shu, Wenwen Xu, Yunzhi Wu

**Affiliations:** 1 Anhui Beidou Precision Agriculture Information Engineering Research Center, Anhui Agricultural University, Hefei 230036, China; wwytt1@stu.ahau.edu.cn (W.W.); dingjie@stu.ahau.edu.cn (J.D.); 22115860@stu.ahau.edu.cn (X.S.); wenwenxu@stu.ahau.edu.cn (W.X.); 2 School of Information and Artificial Intelligence, Anhui Agricultural University, Hefei 230036, China

**Keywords:** pear leaf disease, precise segmentation, FFAE-UNet, attention, feature supplementation

## Abstract

The accurate pest control of pear tree diseases is an urgent need for the realization of smart agriculture, with one of the key challenges being the precise segmentation of pear leaf diseases. However, existing methods show poor segmentation performance due to issues such as the small size of certain pear leaf disease areas, blurred edge details, and background noise interference. To address these problems, this paper proposes an improved U-Net architecture, FFAE-UNet, for the segmentation of pear leaf diseases. Specifically, two innovative modules are introduced in FFAE-UNet: the Attention Guidance Module (AGM) and the Feature Enhancement Supplementation Module (FESM). The AGM module effectively suppresses background noise interference by reconstructing features and accurately capturing spatial and channel relationships, while the FESM module enhances the model’s responsiveness to disease features at different scales through channel aggregation and feature supplementation mechanisms. Experimental results show that FFAE-UNet achieves 86.60%, 92.58%, and 91.85% in MIoU, Dice coefficient, and MPA evaluation metrics, respectively, significantly outperforming current mainstream methods. FFAE-UNet can assist farmers and agricultural experts in more effectively evaluating and managing diseases, thereby enabling precise disease control and management.

## 1. Introduction

Pear trees, as one of the most widely cultivated fruit trees worldwide, have significant economic and ecological value [1]. Their cultivation not only directly affects farmers’ economic returns but also holds an important position in global agricultural production. Pear leaf diseases are key factors impacting pear tree growth and fruit yield, and they are directly related to agricultural productivity and farmers’ income. These diseases can cause leaves to turn yellow and wilt, potentially affect fruit development, and consequently reduce both fruit quality and market value [2]. Therefore, the timely and accurate detection of these diseases is crucial for enhancing agricultural production efficiency.

Traditional disease detection methods rely on experienced farmers or agricultural experts for manual inspection. Although they can identify common diseases, these methods are time-consuming, inefficient, and easily influenced by subjective factors, often resulting in missed detections or misjudgments [3]. This not only affects disease management but can also lead to greater losses. Therefore, there is an urgent need for more efficient and accurate segmentation methods. By using segmentation methods, more detailed disease information can be obtained, and the boundaries of the diseased areas can be delineated to help analyze the morphology and distribution of the disease. This, in turn, facilitates the implementation of more refined disease prevention and control measures.

In the early stages of smart agriculture, traditional machine learning methods identified diseases based on image features (such as color, shape, and texture) [4]. These features were usually manually designed and extracted through a combination of statistical analysis and feature extraction algorithms. Poornima et al. [5] used edge and color features combined with a support vector machine (SVM) for classification; U. Shruthi et al. [6] employed SVM to detect diseases in different crops; Hebbar et al. [7] extracted HoG features and trained them using a random forest classifier to detect leaf diseases; and Dubey et al. [8] adopted K-means clustering to segment lesion areas and combined color and texture features to detect apple diseases. However, because these methods rely on hand-designed features, they often perform poorly in new environments, when facing new diseases, or in complex scenarios, indicating insufficient generalization capabilities.

With the increase in computational power, deep learning has gradually become a growing trend in agricultural production. Compared to traditional machine learning technologies, deep learning stands out in terms of both accuracy and efficiency. Fuentes et al. proposed a real-time tomato plant pest and disease recognition model capable of identifying nine types of tomato diseases [9]. Lu et al. [10] proposed a rice disease identification method based on deep convolutional neural network technology for early disease detection, achieving an accuracy of 95.48%, significantly higher than traditional machine learning models. Lin et al. [11] designed a convolutional neural network-based segmentation model to segment powdery mildew lesions on cucumber leaves, providing a valuable tool for cucumber breeders to assess the severity of powdery mildew.

However, as disease detection tasks become more complex, traditional deep learning models have gradually exposed certain significant limitations. Moreover, when faced with complex backgrounds and diverse disease types, these models often struggle to accurately extract fine-grained features, resulting in inadequate segmentation accuracy. In addition, traditional deep learning models exhibit poor generalization capability when dealing with small disease areas.

To further improve the accuracy and efficiency of segmentation, the encoder–decoder structure has become a widely used architecture. The structure extracts high-level features of the image through the encoder, and then gradually restores the image details through the decoder, which is especially suitable for image segmentation tasks. In agricultural disease recognition, the encoder–decoder architecture can effectively segment different disease regions and show higher robustness when dealing with complex backgrounds and details. Shoaib et al. [12] used U-Net and Modified U-Net to detect and segment tomato disease regions and achieved excellent recognition accuracy. Cui et al. [13] proposed a new network based on ResNet and UNet to effectively extract image segmentation features. Zhang et al. [14] constructed MU-Net for plant disease leaf image segmentation by introducing residual blocks and residual paths, which effectively solved the problem of gradient disappearance and explosion of U-Net and achieved excellent segmentation results.

In order to improve the performance of the model on the above two problems in segmentation tasks, the improvement of feature extraction ability has become the key. In recent years, as an effective means to enhance the ability of feature extraction, the attention mechanism has gradually become an important research direction in the field of image segmentation. By introducing the attention mechanism, the model can better focus on the key disease areas in the image and ignore irrelevant background information, thereby improving the accuracy and robustness of segmentation. Deng et al. [15] proposed cross-layer attention fusion mechanism based on Unet combined with multi-scale convolution module MC-UNet, and achieved good results in tomato leaf disease segmentation. Chen et al. [16] introduced attention mechanism and multi-scale feature fusion into semantic segmentation, which significantly improved the performance of the model in complex scenes. Wang et al. [17] introduced an attention mechanism and a 2-layer 3 × 3 convolution module to optimize the segmentation branch and improve the edge feature representation, and adopted a multi-scale leaf segmentation strategy to obtain the optimal target at different scales. Guo et al. [18] introduced a spatial attention module and adaptively refined the input features so that the network could focus on the key features and improve the representation ability of the network.

However, despite the remarkable progress of the above architectures in many tasks, several challenges remain. For example, it is often difficult for the above structures to maintain high accuracy when dealing with small-scale disease regions or when finer segmentation is required, especially when there is a large difference between the disease region and the background or there is much noise in the image. Moreover, the above methods have insufficient support for feature fusion at different scales, and lack the supplementation and fusion of low-level feature to high-level feature. Therefore, it is necessary to further improve the feature extraction ability of the model, especially when dealing with small disease areas, and enhance the ability to capture detailed information.

In order to deal with these problems, this study proposes a new attention mechanism and adaptive aggregation module, which aims to improve the difficulties and challenges caused by the above problems and further enhance the performance of the model in disease detection. The main work and contributions of this study can be summarized as follows.

1. A novel and efficient pear leaf disease segmentation model FFAE-UNet based on UNet structure is proposed. The model is flexible and efficient, and can adapt to changes in complex backgrounds.
2. The Attention Guiding Module (AGM) is proposed, which combines adaptive channel attention mechanisms, feature fusion and refinement, and multi-view feature selection. This module enhances the model’s focus on disease region features, improving its performance and generalization ability in complex scenarios.
3. The Feature Enhancement and Supplement Module (FESM) is proposed. Different scale convolution and channel aggregation are used to enhance the feature capture ability, and the feature information of the feature map is strengthened to supplement the upsampling feature map, which improves the model’s ability to perceive the edges and small features of the disease.
4. Through comprehensive comparative experiments and quantitative analysis, the FFAE-UNet model has been shown to exhibit superior performance in terms of accuracy, robustness, and adaptability to complex scenarios.

## 2. Materials

### 2.1. Dataset Sources

The pear leaf disease image dataset used in this study includes rust disease, curl disease and disease caused by slugs. Specifically, the disease caused by slugs are labeled as slug. The data mainly come from Dangshan County, Suzhou City, Yingshang County, Fuyang City and Tianmen Town, Tongling City, Anhui Province. In the orchard environment, there are uncontrollable natural factors such as leaf overlap, occlusion, and illumination changes at different angles. In order to comprehensively capture the different poses of leaves, we used a variety of shooting methods to record images from multiple angles. Due to the small size of the Dataset, in order to further enrich the data, we selected images containing rust, curl and slug damage from the public Plant disease image dataset DiaMOS Plant Dataset (Fenu_Malloci_2021) [19] for expansion. The image selection process was tightly controlled to ensure the validity of the experiment, providing a solid and extensive dataset for subsequent research.

### 2.2. Data Processing and Enhancement

To facilitate the deep learning network’s learning on this dataset and meet the requirements of the segmentation tasks, we incorporated data augmentation techniques to further enrich the dataset, including the following:

1. Image rotation, which increases the diversity of the training set by rotating images at random angles.
2. Image brightness adjustment, which randomly changes the saturation, contrast, brightness and sharpness of the image to simulate the disease characteristics in different environments.
3. Random Gaussian noise images are augmented by adding Gaussian noise with a certain probability to enhance the model’s adaptability in complex scenarios.

**Figure 1:**
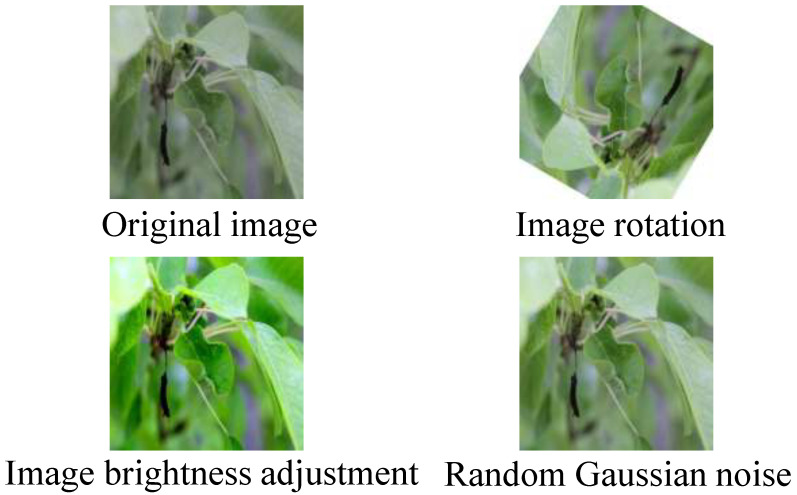
Examples of data augmentation results.

Figure 1 illustrates the pear leaf disease images after data augmentation, which enhances the model’s ability to perform the segmentation task more effectively.

In order to effectively train the segmentation model, Labelme software (version 3.16.7) was used to accurately label the disease parts of pear leaves. With this annotation method, we were able to define the lesion region more accurately and thus improve the performance of the model in the disease segmentation task. The number of datasets formed by our data collection, expansion, and enhancement, as well as the corresponding labeling results, are shown in Table 1.

## 3. Methods

### 3.1. Overall Architecture of FFAE-UNet

**Figure 2:**
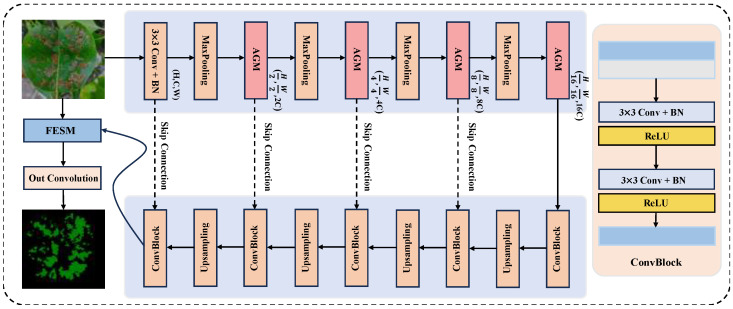
The overall architecture of FFAE-UNet.

In this study, we propose FFAE-UNet, a model based on U-Net [20] architecture, to achieve accurate and efficient segmentation of pear leaf diseases. The overall architecture of FFAE-UNet is shown in Figure 2. The innovation of this model is mainly reflected in two core modules; in the figure, we use red and blue to illustrate the two proposed modules, while the original structure is drawn using a different color. Firstly, we replace the downsampling module of the traditional U-Net with the Attention Guiding Module (AGM) containing the attention mechanism so that the network can more fully obtain the feature information, especially for the details and edges. Secondly, we propose the Feature Enhancement and Supplement Module (FESM) for feature extraction supplement. The edge and small features of the disease area are extracted by channel aggregation and multi-scale convolution so that the network can efficiently fuse and reconstruct features. FFAE-UNet integrates the attention mechanism and feature fusion module, focusing on improving the accuracy and robustness of image segmentation tasks. The proposed network can extract and fuse multi-scale and multi-type features more efficiently, which is especially suitable for pear leaf disease image segmentation tasks with complex backgrounds. It can effectively capture and segment the lesion area in the image, and is sensitive to its boundaries and details.

### 3.2. AGM Module

**Figure 3:**
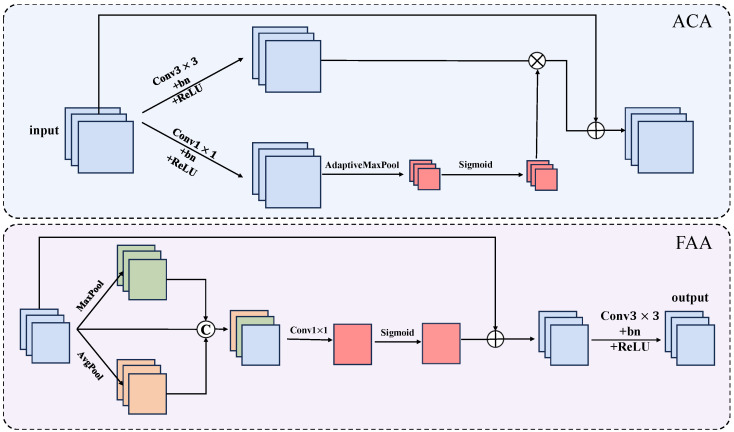
AGM module architecture.

Leaf overlap, branch occlusion and variable illumination in the image make it difficult to segment the disease part. The attention mechanism can make the model focus on and enhance the effective structural features, and ignore part of the useless information so as to improve the stability and robustness of the model to environmental changes. To this end, we propose an Attention Guidance Module (AGM), which is composed of an adaptive channel attention (ACA) part and Feature Aggregation Attention (FAA) part. As shown in Figure 3. By introducing the AGM module, the network’s ability to perceive pear leaf disease is enhanced, allowing it to better capture disease feature information and generate attention maps that are sensitive to both channel and spatial dimensions. This enables more effective feature processing and reconstruction, accurately capturing spatial and channel relationships, and facilitates the handling of details and edges while effectively rejecting irrelevant background elements.

#### 3.2.1. The ACA Part in the AGM Module

ACA operates in the downsampling phase. The core idea of ACA is to adjust the importance of input features based on feature information, enhancing the network’s focus on pear leaf disease regions, especially in the presence of background noise. The description of ACA is as follows: First, the input feature map $X\in R^{H\times W\times C}$ undergoes a 3×3 convolution, batch normalization, and activation by the ReLU function. The process can be expressed as

(1) $$F=\mathrm{ReLU}(\mathrm{Norm}\left({\mathrm{Conv}}_{3}\left(X\right)\right)),$$

where ${\mathrm{Conv}}_{3}$ represents the 3×3 convolution operation, Norm represents batch normalization, ReLU is the activation function, and $F\in R^{H\times W\times C}$.

Simultaneously, the input feature map $X\in R^{H\times W\times C}$ undergoes a 1×1 convolution, batch normalization, and ReLU activation to integrate global information along the channel dimension. Through the adaptive max pooling layer, the global feature $X_{\mathrm{AdMax}}$ is obtained, enabling the reorganization of feature information. The attention weights α are generated using the Sigmoid activation function to represent the importance of different feature channels, selectively amplifying or suppressing specific channels and focusing on important spatial regions of the feature information. This operation helps the network suppress irrelevant features and strengthen its focus on the disease regions, effectively handling the cluttered environments in pear leaf images. Then, the feature map *F* is weighted by the attention weights α, and the result is added to the input feature *X* to supplement the original input information, preventing over-focus. This enhances the prominence of edge regions in the disease feature map while reducing interference caused by redundant information. The process can be expressed as

(2) $$X_{\mathrm{AdMax}}=\mathrm{AMP}\left(\mathrm{Norm}\left({\mathrm{Conv}}_{1}\left(X\right)\right)\right),$$

(3) $$\alpha =\sigma (X_{\mathrm{AdMax}}),$$

(4) $$X_{\mathrm{ACA}}=F⊙\alpha +X.$$

where ${\mathrm{Conv}}_{1}$ represents the 1×1 convolution operation, AMP represents the adaptive max pooling operation, $X_{\mathrm{AdMax}}\in R^{1\times 1\times C}$, σ denotes the Sigmoid activation function, and ⊙ represents element-wise multiplication.

#### 3.2.2. The FAA Part in the AGM Module

The FAA module operates on the output feature map of the ACA module, enhancing the attention mechanism’s ability to perceive spatial dimensions. The FAA module enables the network to capture the prominent disease regions in pear leaf images, helping the network focus on discriminative features, which is crucial for accurately segmenting smaller or less obvious disease areas. The FAA module first processes the received feature map $X_{\mathrm{ACA}}$ through two parallel max pooling and average pooling operations along the spatial dimension to capture the salient regions of the feature map and provide global information. Since pooling operations tend to lose substantial spatial information, the received features $X_{\mathrm{ACA}}$, max pooling features $X_{\max}$, and average pooling features $X_{\mathrm{avg}}$ are concatenated along the channel dimension to achieve a more comprehensive fusion of different features. This helps to better perceive important regions in the image, control the loss of feature information, emphasize discriminative features, and suppress non-discriminative features. The process can be expressed as

(5) $$X_{\max}=\mathrm{Maxpool}\left(X_{\mathrm{ACA}}\right),$$

(6) $$X_{\mathrm{avg}}=\mathrm{Avgpool}\left(X_{\mathrm{ACA}}\right),$$

(7) $$F_{\mathrm{cat}}=\mathrm{Concat}\left(X_{\max},X_{\mathrm{avg}},X_{\mathrm{ACA}}\right).$$

where Maxpool and Avgpool represent max pooling and average pooling operations, respectively, and Concat denotes the concatenation operation.

Next, the concatenated feature $F_{\mathrm{cat}}$ undergoes a 1×1 convolution operation to adjust the channel number of the concatenated feature map. A spatial attention weight β is then generated using the Sigmoid activation function. Subsequently, we weight the feature $F_{\mathrm{cat}}$ using the spatial weight β, enhancing the focus on salient features and desired regions. A convolution operation is then performed to further strengthen the extracted feature information and adjust the output channel number for subsequent operations. The process can be expressed as

(8) $$\beta =\sigma ({\mathrm{Conv}}_{1}\left(F_{\mathrm{cat}}\right)),$$

(9) $$X_{\mathrm{FAA}}=\beta ⊙F_{\mathrm{cat}}+F_{\mathrm{cat}},$$

(10) $$F_{\mathrm{out}}=\mathrm{ReLU}\left(\mathrm{Norm}\left({\mathrm{Conv}}_{3}\left(X_{\mathrm{FAA}}\right)\right)\right).$$

where ${\mathrm{Conv}}_{1}$ represents the 1×1 convolution operation, σ denotes the Sigmoid activation function, ⊙ represents element-wise multiplication, $X_{\mathrm{FAA}}\in R^{H\times W\times 2C}$,${\mathrm{Conv}}_{3}$ represents the 3×3 convolution operation, Norm represents batch normalization, and ReLU is the activation function.

The AGM module enables the network to effectively detect and accurately locate the disease area of pear leaves in a complex environment.

### 3.3. FESM Module

**Figure 4:**
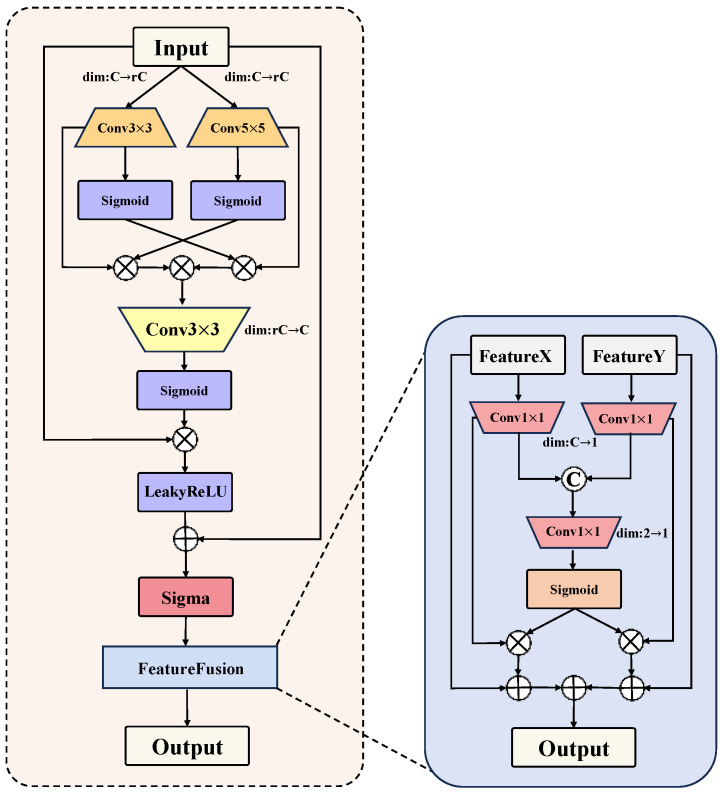
FESM module architecture.

The traditional U-Net is based on an encoder–decoder structure. However, its architecture lacks a feature enhancement and supplementation module, resulting in insufficient response to disease features of different scales, particularly in capturing the edges and fine features of diseased areas. To address these issues, the FESM module is proposed and applied to the pear leaf disease segmentation task. It learns and extracts features at different levels from pear leaf disease images through linear transformations. The final upsampled feature map is then fused with the enhanced feature map through interactive fusion and weight adjustment, achieving feature supplementation between the feature enhancement module and the upsampling module along the spatial dimension. This improves the model’s ability to perceive and segment pear leaf disease lesions as shown in Figure 4.

First, the original input image $X\in R^{H\times W\times C}$ undergoes multi-scale convolution to change its channel dimensions, thereby broadening the receptive field and more comprehensively reflecting disease features. Sigmoid activation is applied to generate attention weights for different convolutions. The outputs of the branches are fused through cross-weight multiplication to enhance the expression of disease feature information. The process can be expressed as

(11) $$F_{1}={\mathrm{Conv}}_{3}\left(X\right),$$

(12) $${\mathrm{Atten}}_{1}=\sigma \left(F_{1}\right),$$

(13) $$F_{2}={\mathrm{Conv}}_{5}\left(X\right),$$

(14) $${\mathrm{Atten}}_{2}=\sigma \left(F_{2}\right),$$

(15) $$F_{\mathrm{mix}}=\left(F_{1}⊙{\mathrm{Atten}}_{2}\right)⊙\left(F_{2}⊙{\mathrm{Atten}}_{1}\right).$$

where ${\mathrm{Conv}}_{3}$ and ${\mathrm{Conv}}_{5}$ represent 3×3 and 5×5 convolution operations, respectively, σ is the Sigmoid activation function, $F_{1}\in R^{H\times W\times rC}$, $F_{2}\in R^{H\times W\times rC}$, *r* is the scaling factor, and ⊙ denotes element-wise multiplication. This process helps the model capture disease feature information across multiple scales and strengthens feature representation through the cross-attention mechanism.

Next, the feature map $F_{\mathrm{mix}}$ is processed by convolution to compress its channel dimensions, thereby collecting and redistributing channel information. The new feature map is activated by the Sigmoid function to generate weights. The initial image is multiplied by the generated weights to obtain the feature map $F_{p}$, and the initial image is added to $F_{p}$ and activated. A learnable scaling coefficient dynamically adjusts the weights and updates the parameters of $F_{\mathrm{add}}$ element-wise. Residual connections are used to alleviate the vanishing gradient problem. The process is as follows:

(16) $$F_{p}=\sigma \left({\mathrm{Conv}}_{3}\left(F_{\mathrm{mix}}\right)\right)⊙X,$$

(17) $$F_{\mathrm{add}}=\mathrm{LeakyReLU}\left(F_{p}+X\right),$$

(18) $$F_{E}=\delta \left(F_{\mathrm{add}}\right)+X.$$

where ${\mathrm{Conv}}_{3}$ is the 3×3 convolution operation, σ is the Sigmoid activation function, ⊙ denotes element-wise multiplication, LeakyReLU is the Leaky ReLU activation function [21], δ is a learnable scaling coefficient, $F_{p}\in R^{H\times W\times C}$, and $F_{\mathrm{add}}\in R^{H\times W\times C}$.

Next, the extracted features $F_{E}$ are fused with the last upsampled layer $F_{U}$ to allow low-level features to supplement high-level features. A convolution operation adjusts the channel dimensions, followed by concatenation along the channel dimension. A second convolution is then applied, and an activation function generates a weight matrix to achieve effective feature fusion. Finally, the weighted compressed features are added to the input feature map. This enhances the model’s ability to integrate multi-level features. The process is as follows:

(19) $$F_{W1}=\mathrm{Norm}\left({\mathrm{Conv}}_{1}\left(F_{E}\right)\right),$$

(20) $$F_{W2}=\mathrm{Norm}\left({\mathrm{Conv}}_{1}\left(F_{U}\right)\right),$$

(21) $$W=\sigma ({\mathrm{Conv}}_{1}\left(\mathrm{Concat}\left(F_{W1},F_{W2}\right)\right)),$$

(22) $$F_{\mathrm{out}}=\left(W⊙F_{W1}+F_{E}\right)+\left(W⊙F_{W2}+F_{U}\right).$$

where ${\mathrm{Conv}}_{1}$ is the 1×1 convolution operation, Norm represents batch normalization, σ is the Sigmoid activation function, Concat is the concatenation operation, ⊙ denotes element-wise multiplication, $F_{W1}\in R^{H\times W\times 1}$, $F_{W2}\in R^{H\times W\times 1}$, $W\in R^{H\times W\times 1}$, and $F_{\mathrm{out}}\in R^{H\times W\times C}$. In this process, through weight adjustment and channel weighting, the model can more precisely integrate low-level and high-level features, thereby enhancing its ability to perceive pear leaf disease lesions.

By integrating the FESM module into the input image, multi-scale features are extracted, enhancing the understanding and processing of features at different scales in complex disease images. Through enhanced channel feature information, the model highlights the importance of different features along the channel dimension. This enhancement effectively avoids feature information blurring, significantly improving the model’s perception ability, especially in handling fine disease features. Furthermore, fusion weights enable more precise adjustment of the proportion of each feature during the fusion process, improving the model’s perception and segmentation capability for diseases.

### 3.4. Loss Functions

During the optimization process, we combine cross-entropy loss with Dice loss as the loss function. This combination not only enhances the model’s ability to recognize and locate pear leaf disease spots in complex environments but is also particularly effective in addressing the issue of class imbalance. Dice loss directly optimizes the overlap between the predicted and true regions, reducing the model’s bias when handling minority classes, thereby improving the model’s performance in segmenting small disease areas. The losses are defined as follows:

(23) $$L_{1}=-\sum \left(t\cdot \log\left(y\right)\right),$$

(24) $$L_{2}=1-\frac{2\cdot \sum (y\cdot t)+s}{\sum y^{2}+\sum t^{2}+s},$$

(25) $$\mathrm{Loss}=\mu \cdot L_{1}+\lambda \cdot L_{2}.$$

where *t* represents the label, *y* is the model’s predicted output, *s* is a hyperparameter set to $1\times 10^{-5}$, and μ and λ are weight coefficients set to 1 and 0.5, respectively.

## 4. Result

### 4.1. Experiment Setting

To ensure the fairness of the evaluation and conduct a systematic comparison between FFAE-UNet and other network architectures, all tests were performed in a unified experimental environment. The experiments were implemented using PyTorch and ran on an NVIDIA 4090 GPU. The training environment configuration is shown in Table 2. The pear leaf dataset was randomly partitioned into training, test, and validation sets in an 8:1:1 ratio. Through various data augmentation methods, a total of 3100 images were generated. The number of diseased leaf samples for each category is listed in Table 1.

During the training process, we trained the model for 100 epochs. Additionally, the batch size was set to 8. The learning rate was adjusted between 0.0001 and 0.000001 based on the training process. We selected Adam as the optimizer, with a momentum of 0.9. Adam combines the optimization strategies of RMSProp and Momentum, enabling adaptive learning rate adjustments to reduce gradient oscillations while accelerating model convergence through the momentum mechanism.

### 4.2. Evaluation Metrics

We used four commonly used evaluation metrics—Mean Intersection over Union (mIoU), Mean Precision (MP), Mean Pixel Accuracy (MPA), and Dice coefficient—to assess the model’s performance in the leaf lesion segmentation task. Below are the definitions of these metrics.

Mean Intersection over Union (MIoU): The MIoU value is an important metric for measuring image segmentation accuracy, representing the average ratio of the intersection and union of the true labels and predicted values for a given class. It is defined as

(26) $$\mathrm{MIoU}=\frac{1}{k+1}\sum_{i=0}^{k}\frac{p_{ii}}{\sum_{j=0}^{k}p_{ij}+\sum_{j=0}^{k}p_{ji}-p_{ii}},$$

where *k* denotes the number of disease categories, *i* represents the ground truth, *j* represents the predicted value, $p_{ij}$ indicates the number of pixels in class *i* predicted as class *j*, $p_{ji}$ and $p_{ij}$ represent false positives and false negatives, respectively, and $p_{ii}$ is the number of true positives.

Mean Pixel Accuracy (MPA) evaluates the pixel-level prediction accuracy of the model, representing the proportion of correctly classified pixels to the total number of pixels. It is defined as

(27) $$\mathrm{MPA}=\frac{1}{N}\sum_{i=1}^{N}\frac{{\mathrm{TP}}_{i}}{{\mathrm{TP}}_{i}+{\mathrm{FN}}_{i}},$$

where *N* represents the number of disease categories detected, TP is the number of true positives, and FN is the number of false negatives.

The Dice coefficient measures the similarity between the predicted results and the ground truth. It is defined as

(28) $$\mathrm{Dice}=\frac{2\cdot |A\cap B|}{\left|A|+|B\right|}$$

where *A* represents the predicted region, and *B* represents the ground truth region.

In addition to these performance metrics, we also evaluated the model’s parameter count and frames per second (FPS) to assess the computational demand and efficiency of the model.

### 4.3. Comparison of Pixels and Base Channels

In Table 3, we detail the specific impact of different input image pixels on the segmentation performance of FFAE-UNet. Through experiments, we compared various pixels settings to evaluate the balance between accuracy, computational burden, and segmentation effectiveness. The results show that the input image pixels directly affects the segmentation accuracy of the model, particularly in recognizing edge details and small-scale diseased areas.

For example, the model’s MIoU at pixels of 128 × 128, 224 × 224, and 256 × 256 are 82.03%, 83.64%, and 84.35%, respectively. While the segmentation performance declines at lower pixels, despite the significant reduction in computational cost, this might be due to the loss of detail information leading to reduced segmentation accuracy. At medium and high pixels settings (e.g., 384 × 384 and 448 × 448), the model achieves better segmentation results with balanced computational efficiency, capturing details accurately while maintaining controlled inference time.

These findings indicate that appropriately increasing the input pixels can significantly enhance the model’s segmentation performance, especially for refined segmentation in boundary regions and small diseased areas.

As shown in Table 4, we configured multiple input convolution feature map channel numbers to further evaluate the impact of initial channel numbers on the segmentation performance of FFAE-UNet. By comparing different channel configurations through experiments, we observed that changes in the feature map channel numbers significantly influence the model’s feature extraction capability, segmentation accuracy, and computational cost.

Lower channel numbers reduce computational cost but may result in insufficient feature representation, thereby affecting segmentation performance. Conversely, higher channel numbers enable richer feature representation, particularly excelling in the recognition of complex diseased areas. The experimental results demonstrate that selecting an appropriate initial feature map channel number achieves an optimal balance between accuracy and efficiency, enabling FFAE-UNet to perform more effectively in pear leaf disease segmentation tasks.

Overall, the choice of pixels and initial channel numbers significantly impacts the model’s computational cost and efficiency. In particular, higher pixels increase inference time, which is especially critical for large-scale datasets or real-time applications. These comparative results provide valuable guidance for selecting pixels in practical applications to achieve an optimal balance between accuracy and efficiency.

### 4.4. Effectiveness Analysis of Individual Modules

In this section, we analyze the impact of specific module adjustments within the model on segmentation performance. The primary evaluation metrics selected are MIoU, MPA, and Dice coefficient.

In Table 5, to validate the effectiveness of the proposed AGM module, we replace it with various commonly used attention mechanisms in the FFAE-UNet and conducted comparative experiments. In the first set of experiments, the AGM was replaced with the ECA module. In the second set, it was substituted with the BAM module. For the third set, the SE module was used in place of AGM. In the fourth set, the GAM module was introduced as a comparison. Finally, in the last set of experiments, the AGM was replaced with the CBAM module.

In each replacement experiment, we evaluated the feature extraction performance of various modules using metrics such as MIoU, cross-entropy loss, and Dice coefficient, and compared them with the FESM module. The replacement experiments validated the effectiveness of the AGM module in the FFAE-UNet model. The experimental results demonstrated that the AGM module significantly outperformed other attention mechanism modules in handling complex backgrounds and detailed features. Its outstanding performance, with an MIoU of 86.60%, MPA of 91.85%, and Dice coefficient of 92.58%, highlights its advantages in enhancing the model’s ability to focus on diseased areas and improve segmentation accuracy.

**Figure 5:**
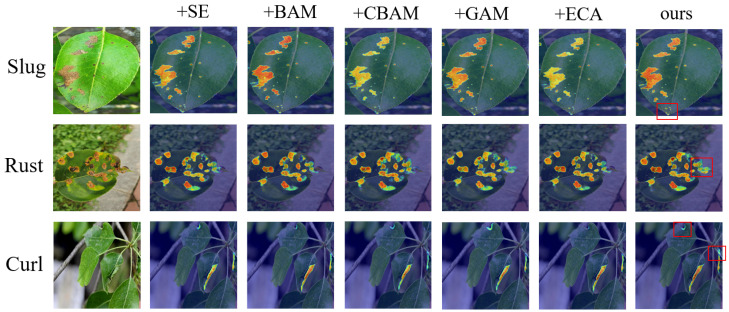
Heatmap visualization of the AGM module compared with other attention modules.

In Figure 5, we perform Grad-CAM [27] heatmap analysis on the models from the replacement experiments and compare them with the FFAE-UNet. In the heatmap, the deeper red areas indicate a higher attention of the model to those regions, reflecting the model’s certainty in identifying the disease areas. Especially in the smaller or more complex disease regions marked by red boxes, FFAE-UNet demonstrates superior segmentation results compared to other models, which show missed detections or lower certainty. For more prominent disease areas, FFAE-UNet shows significantly better attention to the disease regions compared to the models with CBAM, ECA, and SE modules as replacements.

The above experiments demonstrate that incorporating the AGM module leads to better performance in disease boundary localization and handling detailed features. This validates the effectiveness of the proposed module in accurately capturing key features for the pear leaf disease segmentation task.

### 4.5. Comparison with Other Model

In order to verify the model effect of our proposed FFAE-UNet, we compare with some widely used network structures that show excellent performance, as well as architectures also based on UNet. In order to ensure that the experiments are not affected by other factors, all experiments are conducted under a unified benchmark and evaluation index. In the comparative experiments, our proposed network model has obvious advantages in MIoU, MPA, and Dice coefficient. The specific experimental results are shown in Table 6.

**Figure 6:**
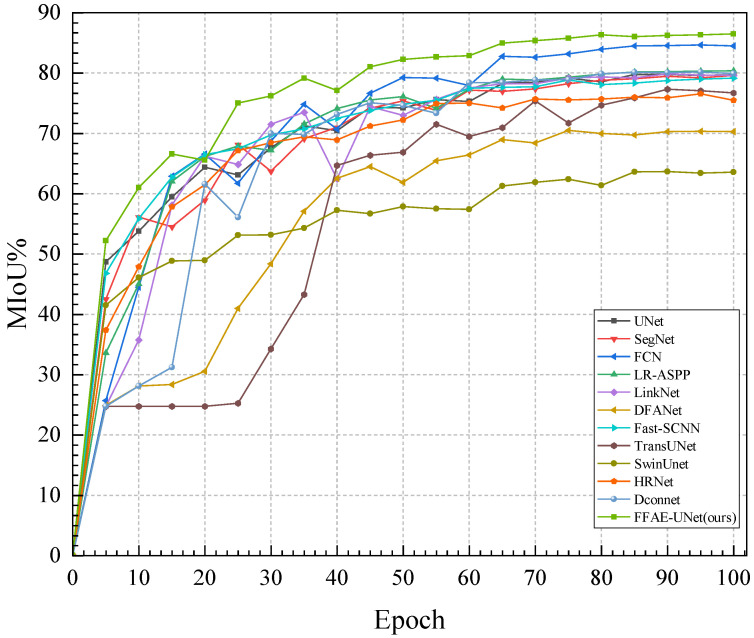
The performance curves of each model on MIoU.

Specifically, on this dataset, the MIoU index of FFAE-UNet reaches 86.50%, which shows that FFAE-UNet has significantly improved the segmentation effect. Compared with other comparative segmentation methods in the experiments, FFAE-UNet has better segmentation performance in almost all other indicators. As shown in Figure 6. The MIoU of Unet, SegNet, FCN, LR-ASPP, LinkNet, DFANet, and Fast-SCNN based on CNN methods are 79.73%, 79.49%, 84.63%, 80.37%, 79.61%, 70.47%, and 78.20%, respectively. The MIoU of TransUNet and SwinUnet based on the Transformer method are 77.43% and 63.57%, respectively. The MIoU of HRNet and Dconnet based on the existing hybrid method are 76.53% and 80.28%, respectively. The improvement in MIoU is primarily due to the attention mechanism introduced in our model, which effectively captures the required features while ignoring irrelevant information. Additionally, the introduced feature enhancement module combines multi-scale convolutions and performs channel aggregation, effectively enhancing detailed features and integrating them with the upsampling results to compensate for the information loss caused by the encoding–decoding process.

The excellent performance of FFAE-UNet in the Average Pixel Accuracy (MPA) and Dice coefficient experiments shows that the proposed model can effectively locate and accurately identify the boundary area of pear leaf diseases. In contrast, the segmentation effect of other models in this dataset is inferior. For example, although DconnNet, LR-ASPP and TransUNet perform better in their respective types of networks with high MIoU values, there is a certain gap in the MPA and Dice coefficient, which is indicated by MPA values of 88.96%, 88.53%, and 85.33%, respectively. The Dice coefficients are 88.65%, 88.63%, and 86.58%, respectively. In contrast, the MPA and Dice values of our proposed FFAE-UNet reach 91.85% and 92.58%, respectively, showing a significant improvement. The above experimental results fully verify the superior performance of FFAE-UNet in plant disease segmentation tasks.

The parameters and FPS of FFAE-UNet are 22.68 M and 24.75, respectively, which are within an acceptable range, and are much smaller than that of SwinUnet and other models. Through the above experiments, FFAE-UNet is able to achieve the accurate and stable segmentation of pear leaf diseases in complex background environments. Additionally, when handling large-scale complex tasks, it effectively optimizes the use of computational resources, demonstrating good computational efficiency.

**Figure 7:**
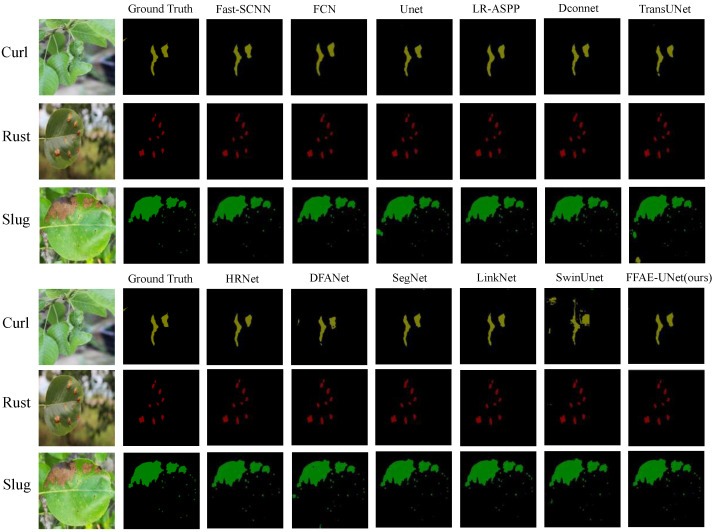
Segmentation results of FFAE-UNet compared with other models.

To comprehensively evaluate the performance of the proposed model in the task of pear leaf disease segmentation, the segmentation results of various models on three different diseases are shown in Figure 7. The yellow markings represent the segmentation results for Curl disease, the red markings indicate the segmentation results for Rust disease, and the green markings correspond to the segmentation results for Slug disease.

From the segmentation results, it can be seen that some networks (such as UNet, SegNet, FCN and DconnNet) have the missegmentation of pear leaf disease regions in the segmentation task; especially when dealing with the vein, shadow, and background junction regions, the disease feature extraction ability is weak. In contrast, our FFAE-UNet can segment the disease region more accurately, and the segmentation results are basically consistent with the actual disease distribution. Compared with other networks such as UNet, FFAE-UNet performs well in distinguishing the edge area of the disease from the shadow part, and can achieve accurate segmentation even in complex backgrounds.

In addition, FFAE-UNet can effectively retain semantic information through multi-scale feature extraction, enhance the capture of detailed features, and enrich the feature information of the network when dealing with uncontrollable natural factors such as leaf overlap, occlusion, and illumination changes at different angles. Experimental results show that FFAE-UNet can effectively focus on the disease area by feature enhancement in the channel dimension and spatial dimension respectively. Multi-scale feature capture further improves the model’s ability to perceive the target, especially in the segmentation accuracy of details and edge regions.

**Figure 8:**
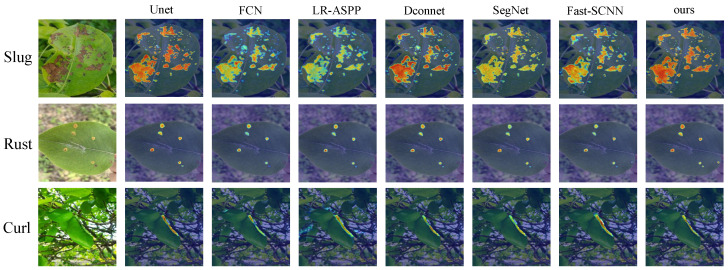
Heatmap visualization of FFAE-UNet compared with selected other models.

To evaluate the performance of the model more clearly, we used Grad-CAM to visualize the attention of the model to the diseased area and generate the corresponding heat map. In Figure 8, we selected the network with a higher Dice coefficient for comparison. Through these visualizations, it is clearly observed that FFAE-UNet shows excellent ability to identify and precisely locate pear leaf disease features.

The model shows excellent accuracy and coverage under a variety of disease types, and especially in the small and dense spots formed by rust disease, FFAE-UNet is more accurate in locating the focus. Thanks to the channel aggregation and multi-scale convolution module in the model, it has high sensitivity to the disease area of complex background. In the curl area of leaf edge caused by leaf curl disease, the performance advantage of FFAE-UNet is more significant, which can accurately highlight the disease area and effectively reduce the interference of background noise. In the case of large disease spots, FFAE-UNet can still accurately focus on the disease site. This is thanks to the adaptive channel and spatial attention mechanism of FFAE-UNet, which can accurately highlight the diseased area while reducing the attention to background noise.

Through these heat map analysis, the design advantage of FFAE-UNet lies in its significant attention and accurate positioning of the disease area, which makes it show higher robustness and detail processing ability in pear leaf disease detection tasks.

### 4.6. Ablations Experiments

**Figure 9:**
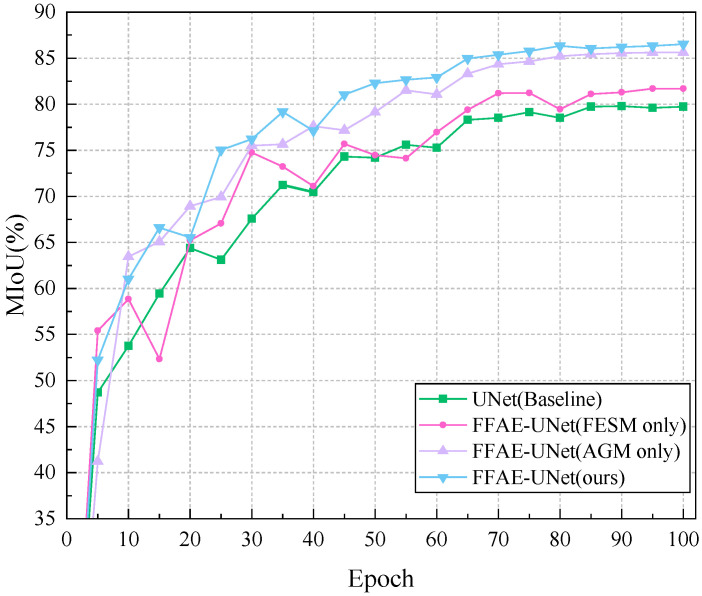
MIoU curves of the ablation experiment results for FFAE-UNet.

In this section, we conduct various ablation experiments to analyze the effectiveness of the proposed modules. We adopt the control variable method and use the standard UNet network as the base network, gradually introducing the Attention Guiding Module (AGM) and Feature Enhancement and Supplement Module (FESM). The result metrics of the experiments are shown in Table 7. We analyze the performance of each module and its importance to the network by analyzing the variation of MPA, MIoU, and Dice coefficient. When AGM is combined with the baseline, MIoU increases by 5.87%, Dice increases by 3.84%, and MPA increases by 2.51%. Figure 9 shows the MIoU change curve of each ablation experiment on the training set in detail. In addition, when FESM module is combined with the baseline, MIoU increases by 1.97% and Dice increases by 1.24%. According to the experimental results, our proposed FFAE-UNet can achieve excellent segmentation results, which indicates that multi-scale feature extraction and adaptive attention mechanism are necessary for pear leaf disease information extraction.

In conclusion, each module of FFAE-UNet positively impacts both MIoU and the Dice coefficient, which confirms that the proposed AGM and FESM modules are effective.

## 5. Discussion

In this study, we propose an improved U-Net-based network architecture, FFAE-UNet, specifically designed for the segmentation of pear leaf diseases. The FFAE-UNet incorporates the Attention Guidance Module (AGM) and Feature Enhancement Supplement Module (FESM) to improve the segmentation accuracy and robustness.

During the model training process, we combined cross-entropy loss and Dice loss to further optimize performance. Cross-entropy loss focuses on overall pixel classification accuracy, while Dice loss emphasizes overlapping regions, enhancing the model’s attention to small targets. This combined loss function strategy improves the model’s ability to capture both boundary details and overall shapes of disease spots in complex environments.

To validate the effectiveness of the proposed modules, we conducted an effectiveness analysis and ablation experiments. The experimental results demonstrate the efficacy of the proposed AGM and FESM modules in enhancing the model’s segmentation performance. Additionally, we tested the proposed network with different base channels and pixels to find the balance between computational efficiency and segmentation performance.

Although advanced segmentation networks such as TransUNet and SwinUNet perform well on standard datasets, they yield suboptimal results when applied to the segmentation of pear leaf disease images. This limitation may stem from challenges such as scale variations and inconsistent imaging conditions inherent in photographs taken in natural environments, which pose adaptation challenges for these models.

Whereas the number of parameters of the proposed model is higher than LR-ASPP, DFANet, and Fast-SCNN, the number of parameters is relatively small compared to other more commonly used models in this field. For example, it is significantly smaller than models such as Swin-Unet and TransUNet. In terms of performance indicators, the segmentation effect of FFAE-UNet is greatly improved, and the visual segmentation map and heat map clearly reflect the superior performance of the model for disease region segmentation.

FFAE-UNet demonstrates superior performance in the current task. However, we acknowledge that future research should focus on further optimizing the network architecture to better accommodate more complex environmental variations and further improve the model’s robustness and segmentation accuracy.

## 6. Conclusions

This study proposes a network specifically designed for pear leaf disease segmentation, named FFAE-UNet. Based on the U-Net architecture, FFAE-UNet comprises encoding and decoding stages, which enable the capture and preservation of multi-scale contextual information in images. We introduce the AGM and FESM modules to enhance the network’s performance.

The AGM module employs an adaptive channel attention mechanism to dynamically adjust the weights of feature channels, significantly improving the model’s focus on key diseased regions while effectively suppressing background noise. This allows the model to more accurately extract disease features in complex scenarios. The FESM module aggregates channel features to extract rich detailed information and employs a feature supplementation mechanism that integrates upsampled feature maps with enhanced feature maps through interactive fusion. By dynamically adjusting weights along the spatial dimension, FESM strengthens the connection between the enhancement module and the upsampling module, thereby substantially improving segmentation precision in small-scale diseased areas and edge details.

Under identical experimental settings, FFAE-UNet outperformed current state-of-the-art segmentation methods. Specifically, FFAE-UNet achieved 86.60%, 92.58%, and 91.85% in MIoU, Dice coefficient, and MPA, respectively.

The success of this method demonstrates significant application potential in precision agriculture and crop health management. By precisely segmenting diseased areas on pear leaves, FFAE-UNet enables early disease detection, helping farmers implement targeted prevention measures to prevent disease spread and enhance control effectiveness.

In future work, we aim to further improve the processing efficiency of FFAE-UNet and explore its potential applications in a broader range of scenarios.

## Figures and Tables

**Table 1:** Dataset structure display.

Image	Number (Before)	Number (After)	Proportion (%)
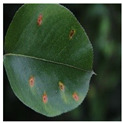	1046	1046	33.7
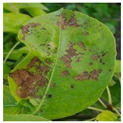	461	972	31.3
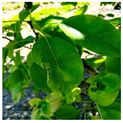	157	1082	34.9

**Table 2:** Training environment setting.

Category	Component	Specification
Hardware environment	CPU	Intel(R) Core(TM) i9-13900K
	GPU	NVIDIA GeForce RTX 4090
	RAM	32 G
Software environment	Torch	1.8.1
	Python	3.8.8
	CUDA Toolkit	V11.3

**Table 3:** Comparison of effects at different image pixels.

Pixels	MIoU	MPA	Dice
128 × 128	82.03%	89.61%	89.74%
224 × 224	83.64%	91.33%	90.80%
256 × 256	84.35%	89.58%	91.26%
384 × 384	84.47%	89.80%	90.93%
448 × 448	85.79%	91.11%	92.14%
512 × 512	86.60%	91.85%	92.58%

**Table 4:** Comparison of effects with different base channels.

Channels	MIoU	MPA	Dice	GFlops (G)
16	79.40%	87.19%	87.90%	11.60
32	82.93%	88.78%	90.35%	44.39
64	86.60%	91.85%	92.58%	174.81

**Table 5:** Comparison experiments of the AGM module in FFAE-UNet with other attention modules.

Model	MIoU	MPA	Dice	FPS
+SE [22]	83.60%	91.04%	90.78%	21.54
+BAM [23]	82.73%	89.49%	90.23%	21.18
+CBAM [24]	85.13%	89.95%	91.73%	19.56
+GAM [25]	85.02%	90.13%	91.67%	19.72
+ECA [26]	84.02%	90.38%	91.06%	21.58
FFAE-UNet	86.60%	91.85%	92.58%	24.75

**Table 6:** Performance comparison among different models.

Model	MIoU	MPA	Dice	FPS	Parameters
UNet [20]	79.73%	88.52%	88.18%	28.22	7.08 M
SegNet [28]	79.49%	87.16%	88.07%	37.91	29.44 M
FCN [29]	84.63%	90.18%	91.44%	20.83	32.94 M
LR-ASPP [30]	80.37%	88.53%	88.63%	123.14	3.21 M
LinkNet [31]	79.61%	87.98%	88.13%	86.41	11.53 M
DFANet [32]	70.47%	81.37%	80.94%	36.30	2.16 M
Fast-SCNN [33]	78.20%	88.03%	87.08%	109.64	1.13 M
TransUNet [34]	77.43%	85.33%	86.58%	36.28	63.72 M
Swin-Unet [35]	63.57%	63.57%	82.96%	23.14	41.34 M
HRNet [36]	76.53%	86.84%	85.89%	23.32	29.53 M
Dconnet [37]	80.28%	88.96%	88.65%	46.05	25.84 M
FFAE-UNet	86.50%	91.85%	92.58%	24.75	22.68 M

**Table 7:** Ablation experiments for FFAE-UNet.

NO.	Trick and Methods		MIoU	MPA	Dice
	**AGM**	**FESM**			
1	-	-	79.73%	88.52%	88.18%
2	-	✓	81.43%	88.78%	89.42%
3	✓	-	85.61%	91.03%	92.02%
4	✓	✓	86.60%	91.85%	92.58%

## Data Availability

All data will be provided upon request to the authors.
